# Use of fixed-dose combination antihypertensives in Germany between 2016 and 2020: an example of guideline inertia

**DOI:** 10.1007/s00392-022-01993-5

**Published:** 2022-02-27

**Authors:** Felix Mahfoud, Marita Kieble, Salka Enners, Ulrich Kintscher, Ulrich Laufs, Michael Böhm, Martin Schulz

**Affiliations:** 1 Klinik für Innere Medizin III, Universitätsklinikum des Saarlandes, Kirrberger Str. 1, 66421 Homburg/Saar, Germany; 2 Deutsches Arzneiprüfungsinstitut e. V. (DAPI), Heidestr. 7, 10557 Berlin, Germany; 3 Institut für Pharmakologie, Charité – Universitätsmedizin Berlin, Hessische Str. 3-4, 10115 Berlin, Germany; 4 Klinik und Poliklinik für Kardiologie, Universitätsklinikum Leipzig, Liebigstr. 20, 04103 Leipzig, Germany; 5 Institut für Pharmazie, Freie Universität Berlin, Kelchstr. 31, 12169 Berlin, Germany

**Keywords:** Antihypertensives, Fixed-dose combinations, Drug utilization, Guideline implementation

## Abstract

**Background:**

The 2018 European Society of Cardiology (ESC)/European Society of Hypertension (ESH) guidelines for the management of hypertension highlight the importance of fixed-dose combinations (FDC) for the treatment of hypertension and recommend initial single-pill combination therapy in almost all patients. However, data on the implementation of these recommendations in clinical practice are scarce.

**Methods:**

Data from the *German Institute for Drug Use Evaluation (DAPI)* were analyzed and extrapolated accounting for approximately 88% of Germany’s population (approximately 73.3 million subjects). All antihypertensive (AHT) FDC products available on the German market were included in the analyses. We examined the time course of dispensed packages between January 2016 and December 2020.

**Results:**

FDCs accounted for 15.4% of all AHT in 2016 and for 10.9% in 2020. While dispensing of all AHT increased slightly from year to year (2016: 143.8 million, 2020: 153.2 million packs), dispensing of FDCs decreased from 22.2 million (2016) to 16.6 million (2020) packs. Dispensing of FDCs containing hydrochlorothiazide (HCT) declined considerably from 2016 to 2020 (Q1 2016: 4.65 million, Q4 2020: 3.13 million packs). Accordingly, the proportion of HCT-containing combinations in all FDCs decreased from 85.3 to 74.2% from Q1 2016 to Q4 2020. Patients younger than 80 years were prescribed FDCs more frequently (14.6% of all AHT, based on the entire evaluation period) than patients 80 years and older (10.0%). In both age groups, this proportion decreased continuously over time.

**Conclusions:**

Almost 2 years following the release of the 2018 ESC/ESH guidelines, only 10.9% of the prescribed packs of antihypertensive drugs in 2020 were FDC products, documenting underutilization of current guideline recommendations on pharmacotherapy in hypertension. Structured programs to evidence-based decision support are required to improve guideline inertia and patient outcomes, eventually.

**Graphical abstract:**

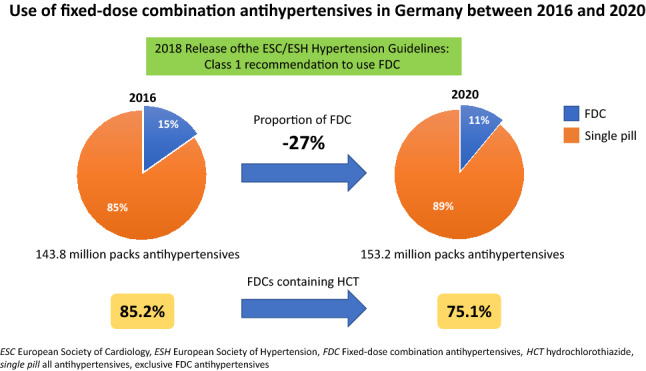

## Introduction

The 2018 European Society of Cardiology (ESC)/European Society of Hypertension (ESH) guidelines for the management of hypertension highlight the importance of fixed-dose combinations (FDC) for the treatment of hypertension and recommend initial single-pill combination therapy in almost all patients [1]. FDCs are also recommended by the Japanese Society of Hypertension (JSH) guidelines to improve medication adherence and blood pressure control [2]. This strategy has been shown to improve medication adherence, tolerability and consequently blood pressure control and associates with lower pill burden and reduced medical costs [1, 3, 4]. However, data on the implementation of these recommendations for the treatment of hypertension in clinical practice are scarce.

## Methods

The analysis is based on data from the *German Institute for Drug Use Evaluation (Deutsches Arzneiprüfungsinstitut e.V., DAPI, *www.dapi.de*).* The database contains anonymized dispensing data from more than 80% (until June 2019) and more than 95% (from July 2019 onwards) of Germany’s community pharmacies, claimed at the expense of the statutory health insurance (SHI) funds. Data were extrapolated by regional factors to 100% of the SHI-insured population, which accounts for approximately 88% of Germany’s population i.e., appr. 73.3 million subjects [3]. All antihypertensive FDC products available on the German market were included in the analyses [5]. As a reference group, all antihypertensive (AHT) drug products were considered (Anatomical Therapeutic Chemical (ATC) classification system: antihypertensive drugs [C02], diuretics [C03], beta blockers [C07], calcium channel blockers (CCB) [C08], ACE inhibitors (ACEi)/angiotensin receptor blockers (ARB) [C09], and statins in combination with antihypertensives [C10BX]).

We examined the time course of dispensed packages between January 2016 and December 2020 on a quarterly (Q) basis for all AHT FDC in total and separately for products with and without hydrochlorothiazide (HCT) as well as by drug classes. Furthermore, we analyzed dispensing in the age groups < 80 vs. ≥ 80 years.

## Results

**Fig. 1:**
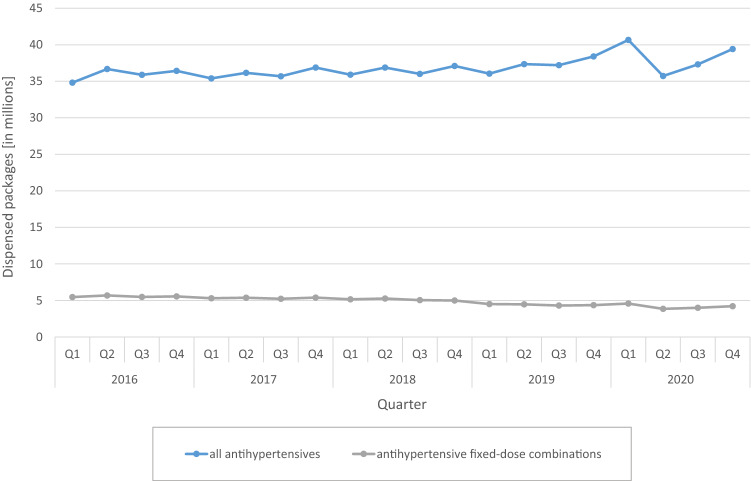
Dispensings of antihypertensives and antihypertensive fixed-dose combination products on a quarterly (Q) basis from 2016 to 2020

FDCs accounted for 15.4% of all AHT in 2016 and for 10.9% in 2020 (Fig. 1). While dispensing of all AHT increased slightly from year to year (2016: 143.8 million, 2020: 153.2 million packs), dispensing of FDCs decreased from 22.2 million (2016) to 16.6 million (2020) packs over the same period.

**Fig. 2:**
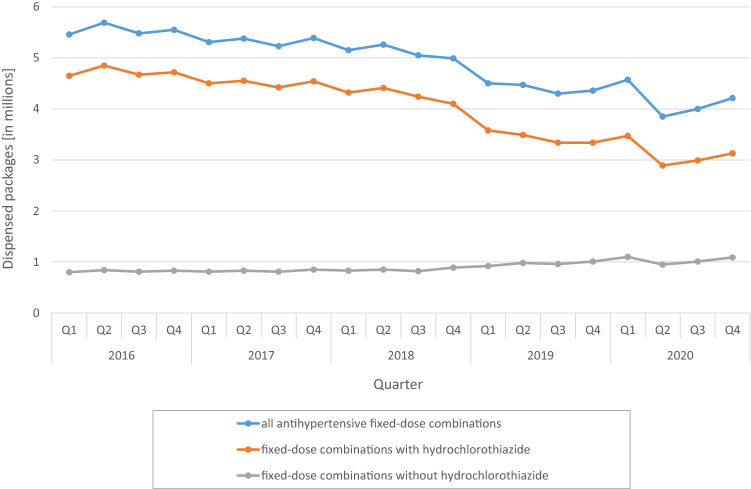
Dispensings of antihypertensive fixed-dose combination products (depending on whether hydrochlorothiazide was contained) on a quarterly (Q) basis from 2016 to 2020

Dispensing of FDCs containing HCT declined slightly from 2016 to 2018 (Q1 2016: 4.65 million, Q4 2018: 4.10 million packs) and then considerably dropped to 3.13 million packs in Q4 2020. In contrast, FDCs without HCT showed a minor increase in dispensing from Q4 2018 onwards (Q4 2018: 0.89 million, Q4 2020: 1.09 million packs). Accordingly, the proportion of HCT-containing combinations in all FDCs decreased from 85.3 to 74.2% from Q1 2016 to Q4 2020 (Fig. 2).

The most frequently dispensed FDCs were ACEi + HCT and ARB + HCT, respectively. Their dispensing initially fell slightly until Q3 2018 and then somewhat more sharply as of Q4 2018 (ACEi + HCT: Q1 2016: 1.84 million, Q4 2018: 1.54 million, Q4 2020: 1.13 million packs; ARB + HCT: Q1 2016: 1.75 million, Q4 2018: 1.67 million, Q4 2020: 1.29 million packs). In contrast, increases in dispensing was observed from Q4 2018 onwards in the third and fourth most common groups of ACEi + CCB (Q4 2018: 0.36 million, Q4 2020: 0.41 million) and ARB + CCB (Q4 2018: 0.36 million, Q4 2020: 0.46 million).

**Fig. 3:**
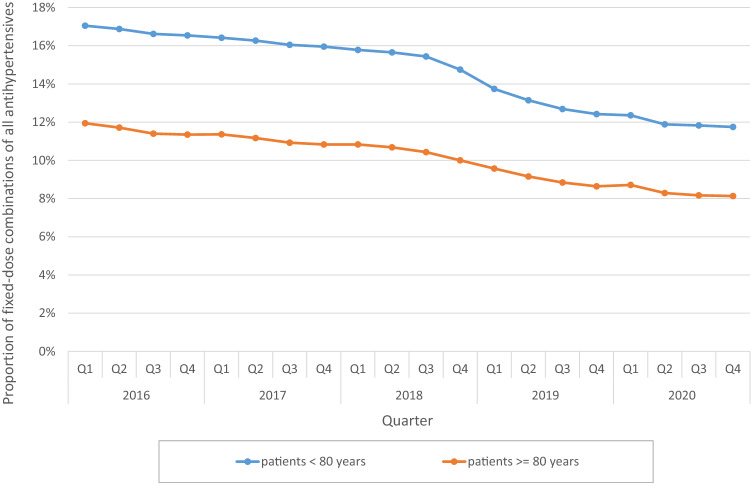
Proportion of fixed-dose combination products of all antihypertensives by age group on a quarterly (Q) basis from 2016 to 2020

The analysis by age group showed that patients younger than 80 years were prescribed FDCs more frequently (14.6% of all AHT, based on the entire evaluation period) than patients 80 years and older (10.0%). In both age groups, this proportion decreased continuously over time (Fig. 3).

## Discussion

A high proportion of patients with hypertension fail to reach guideline recommended blood pressure targets [6]. Among the major causes for insufficient blood pressure control are physician inertia and patient non-adherence to antihypertensive medication [7]. The latter is frequently observed in hypertension treatment and associates with increased morbidity and mortality [7–10]. Fixed-dose combinations were shown to improve adherence and blood pressure control [4]. Hence, the 2018 ESC/ESH guidelines for the management of hypertension recommended initiating an antihypertensive treatment with a two-drug combination in a single-pill combination in most patients. The exceptions include frail or very old patients (> 80 years) and those at low risk with moderate hypertension (particularly if systolic blood pressure is < 150 mmHg) [1]. The current analysis aimed at investigating the utilization of this recommendation in clinical practice in Germany. The major findings are: (i) FDCs accounted only for 15.4% of all AHT in 2016 and for 10.9% in 2020; (ii) FDCs containing HCT declined from 85.3 to 74.2% over the period of investigation, and (iii) FDCs were more frequently prescribed in patients younger than 80 years (14.6% versus 10%).

The use of FDCs in hypertension has been associated with several advantages, including improved medication adherence and persistence, and blood pressure outcomes when compared with free combinations. Of antihypertensive drugs, diuretics are more likely to lead to poor adherence, but adherence can be improved if diuretics are prescribed as fixed-dose combinations [1, 2, 11]. A randomized, controlled trial investigating a once-daily fixed-dose triple combination pill (20 mg telmisartan, 2.5 mg amlodipine, 12.5 mg chlorthalidone) versus usual care in 700 hypertensive patients requiring initiation (untreated patients) or escalation (patients receiving monotherapy) of antihypertensive therapy documented an increased proportion of patients achieving their target blood pressure goal with FDC therapy (70% versus 55%; risk difference 12.7%; 95% CI 3.2–22.0%; *p* < 0.001) [12]. In a recent meta-analysis comprising 44 studies, FDC therapy was associated with significantly larger reductions in both systolic and diastolic blood pressure at week 12 when compared with free combinations (mean difference − 3.99 mmHg; 95% CI, − 7.92 to − 0.07; *p* = 0.05 and − 1.54; 95% CI, − 2.67 to − 0.41; *p* = 0.0076). Rates of adherence and persistence were higher with FDC, which was in turn associated with better BP control [4]. Against the background of solid evidence supporting the use of FDC in hypertension management, the recent ESC/ESH guidelines advise their use in almost all hypertensive patients (Class 1 A recommendation). These recommendations were published back in August 2018 [1] and adopted by the German Cardiac Society/German Hypertension League (DGK/DHL) immediately thereafter in 2019 [13]. Despite these explicit recommendations, we show herein that only a small proportion of all patients in Germany were prescribed FDCs. Interestingly, this number further decreased over the years. The possible reasons for the poor adherence of physicians to comply with guideline recommendations are likely multifactorial, e.g., degree of specialization, work experience, and level of care center [14]. A study in Japan showed an association between the size of health care facilities and the intensity of hypertension therapy. Physicians in larger facilities prescribed higher number of AHT drug classes compared with those working in smaller facilities and clinics [15]. Beside these factors, some statutory health insurance companies in Germany still select payment of single rather than fixed-dose combinations, referring to the guidelines on General Practitioner Risk Advice on Cardiovascular Prevention of the German College of General Practitioners and Family Physicians (DEGAM, valid until 31 December 2021) which have not recommended FDCs but rather free combinations [16]. It is, therefore, plausible that physicians tend to prescribe generic free combinations to avoid recourse claims by payers. We hypothesize that the under use of guideline recommended FDC is one of the reasons for the low blood pressure control rates in Germany [17], recently reported to be only 48% in treated patients [6].

The majority of the approved FDC in Germany contain the diuretic HCT, which is one of the most frequently prescribed medications in Germany [18]. Recently, concerns have been expressed through the publication of a “Dear Doctor Letter” (Rote Hand Brief, Bundesinstitut für Arzneimittel und Medizinprodukte, BfArM) regarding a potential photosensitizing effect (i.e., presumed to be related to the sulfonamide structure) and an increased, dose-dependent risk of skin cancers [19]. The Drug Commission of the German Medical Association (Arzneimittelkommission der Ärzteschaft) subsequently recommended no general therapeutic switch for all patients treated with HCT, but that the indication and risk should be considered individually. In a study, investigating the sales of all antihypertensive substances from January 2017 to December 2019 in Germany, it was documented that dispensings of HCT-containing drugs fell notably. The increase in sales of alternative diuretics (indapamide, chlorthalidone, and others) accounted only for about 22% of the drop in sales of packs of HCT. Hence, it is likely that in approximately 700,000 patients who had previously been treated with HCT, the diuretic component of the antihypertensive treatment scheme was dropped without appropriate replacement [18]. The findings of the present analysis indicate that also FDCs containing HCT declined by 33% over the period of investigation (from 4.65 Mio in Q1/2016 to 3.13 Mio packs in Q4/2020). Since most of the FDCs in Germany contain HCT as the diuretic component, this is among the main reason for the overall reduction in the prescription of FDCs. As there are few alternative FDCs containing diuretics other than HCT available, FDCs with ACEi/ARB and CCB are conceivable alternatives that are recommended in the guidelines as equivalent to FDCs with ACEi/ARB and diuretics. Encouragingly, these FDCs have actually seen an, although slight, increase in prescriptions of 13% for ACEi + CCB and 27% for ARB + CCB since Q4 2018, respectively.

The current ESC/ESH hypertension guidelines recommend that older patients (> 65 years, including patients over 80 years) should be offered blood pressure-lowering treatment if their systolic blood pressure is > 160 mmHg with a target of 130–139 mmHg. This should be achieved with lifestyle modification and, in most cases, antihypertensive drug therapy. In individuals older than 80 years of age, initiation of antihypertensive monotherapy is recommended [1]. In line with this recommendation, the proportion of patients treated with FDC was higher in the age group < 80 years as compared with older individuals (14.6% versus 10% of the dispensed packs with antihypertensives).

## Conclusions

This analysis investigating the utilization of current guideline recommendations on pharmacotherapy in hypertension between 2016 and 2020 documented high rates of guideline non-adherence in Germany. Almost 2 years following the release of the 2018 ESC/ESH hypertension guidelines, only 10.9% of the prescribed packs of antihypertensive drugs in 2020 were FDC products, known to improve medication adherence and blood pressure control. Structured programs to evidence-based decision support are required to improve guideline adherence and patient outcomes, eventually.

## Data Availability

Data are available upon reasonable request by DAPI.
